# Data privacy governance and employee trust: evidence from Vietnamese commercial banks

**DOI:** 10.3389/frma.2026.1910765

**Published:** 2026-08-14

**Authors:** Thanh Vo Phuc Truong, Quoc-Hieu Phan, Cuong Tran Thi, Linh Ha Le Khanh, Hai Tran Xuan Hoang

**Affiliations:** 1 Department of Finance and Banking, School of Economics, Can Tho University, Can Tho, Vietnam; 2 Department of Business Administration, Chaoyang University of Technology, Taichung, Taiwan; 3 Department of Business Management, National Economics University, Hanoi, Vietnam; 4 Independent Researcher, Ho Chi Minh City, Vietnam; 5 Faculty of Business Administration and Marketing, Hung Vuong University of Ho Chi Minh City, Ho Chi Minh City, Vietnam

**Keywords:** commercial banks, digital ethics, digital transformation, employee trust, organizational justice

## Abstract

**Introduction:**

This study examines the structural mechanisms through which digital ethics governance and data protection strategies influence employee trust and adaptive performance in the commercial banking sector.

**Methods:**

A cross-sectional quantitative design was employed, collecting data from 402 bank employees in Vietnam. Partial Least Squares Structural Equation Modeling (PLS-SEM) and PLSpredict were used to test the hypotheses and evaluate predictive capabilities.

**Results:**

Results reveal that digital ethics governance and data protection significantly enhance organizational justice. Organizational justice acts as a critical mediator toward employee trust. Furthermore, digital transformation intensity and organizational culture support directly promote employee trust, which in turn drives adaptive behavior.

**Discussion:**

The findings highlight that technological adoption alone is insufficient without strong ethical governance and structural support. Banks must integrate ethical safeguards with culture and justice mechanisms to foster trust and adaptive performance.

## 1 Introduction

Vietnam's banking sector is undergoing rapid and large-scale digital transformation. The number of digital banking users, technology investment levels, and the rate of business process digitization have all grown substantially in recent years (State Bank of Vietnam, 2021, 2024). However, alongside technological advances, many banks continue to face a gap between the capabilities of deployed systems and actual employee utilization. Research consistently shows that digital transformation failures are more often attributable to human factors than to technological constraints (McKinsey & Company., 2023). This underscores employee trust as a critical determinant of digital transformation effectiveness in organizations.

In this context, bank employees must adapt to changes involving digital workflows, digital monitoring, and data privacy. They are expected to use new digital systems while simultaneously confronting concerns about personal data control, the transparency of automated decisions, and organizational accountability. Digital transformation is therefore not merely a technological matter but a process of restructuring the relationship between organizations and employees, with trust at its center.

Yet the mechanism through which employee trust is formed under digital transformation remains incompletely understood. The Privacy Calculus Theory posits that perceived risks to personal data reduce acceptance of digital systems (Dinev and Hart, 2006). Conversely, Social Exchange Theory emphasizes that trust is built when organizations demonstrate fairness, respect, and accountability in governance (Blau, 1964). Meanwhile, leadership research in high power-distance contexts highlights employees' reliance on managerial signals to interpret organizational change. These perspectives are closely related but rarely tested simultaneously.

The Vietnamese context is particularly suitable for examining these issues. The enactment of Decree 13/2023/ND-CP on Personal Data Protection has substantially elevated privacy awareness among the workforce (Vietnamese Government, 2023). At the same time, collectivist cultural characteristics and high power distance may modify how employees respond to data governance, organizational justice, and leadership behavior compared to Western contexts. Yet most existing research on Vietnamese banking digitization focuses on financial performance, risk management, or customer behavior, leaving the employee perspective and internal data governance relatively underexplored (Do et al., 2022; Nguyen et al., 2024; Hoang et al., 2023). A recent conceptual analysis of digital ethics in banking (Bahzad and Taleb, 2026) likewise emphasizes that ethical practice, not merely technological capability, is what ultimately sustains trust and performance gains from digital transformation, reinforcing the relevance of an employee-facing, ethics-centered lens for the present study.

To address this gap, the present study develops an integrated model explaining employee trust in the context of bank digital transformation. Specifically, it tests the impact of digital ethics governance on employee trust, the mediating role of organizational justice perceptions, and the moderating role of individual digital capabilities. In doing so, the study contributes to the literature on digital transformation, organizational trust, and data governance by providing empirical evidence from the Vietnamese banking context in the post-Decree 13/2023 era.

## 2 Theoretical background and hypotheses

### 2.1 Integrated theoretical framework

The study constructs an integrated theoretical framework to comprehensively explain the formation of digital transformation behaviors among banking employees. To explain organizational-level factors, the study draws on the Resource-Based View (Barney, 1991) combined with Organizational Climate Theory (Schneider et al., 2013). Digital transformation capability depends not only on tangible technological resources but also on strategically valuable intangible resources such as leadership support and organizational culture. Building on this foundation, Social Exchange Theory (Blau, 1964) explains that when organizations demonstrate responsibility through digital ethics governance, data protection, and fair treatment, employees perceive reciprocity and form trust, responding with positive behaviors (Cropanzano and Mitchell, 2005). Additionally, the Privacy Calculus Model (Dinev and Hart, 2006) and the Technology Acceptance Model (Davis, 1989; Venkatesh and Davis, 2000) emphasize trust as a critical psychological antecedent leading to effective technology acceptance, particularly in data-sensitive contexts (Gefen et al., 2003).

Given the breadth of theories invoked above, Social Exchange Theory (Blau, 1964) is treated as the primary theoretical anchor of the model, since the core causal chain from governance practices through justice perceptions to trust and behavioral response is fundamentally a reciprocity mechanism. The remaining theories play clearly bounded supporting roles: the Resource-Based View and Organizational Climate Theory motivate the organizational-level antecedents (DT intensity, organizational culture, leadership support, digital readiness) as strategic resources; the Privacy Calculus Model and Technology Acceptance Model motivate the individual-level psychological mechanism linking trust to system-related behavior; and Organizational Justice Theory specifies the mediating construct through which reciprocity is cognitively processed. This division of theoretical labor keeps the framework anchored rather than fragmented across six co-equal perspectives.

### 2.2 Digital ethics governance, data protection, and organizational justice

Digital ethics is defined as an organization's commitment to using data and technology transparently, impartially, and accountably (Shaikh et al., 2025). According to social exchange theory, these ethical actions signal organizational respect and responsibility, thereby reinforcing justice perceptions. Greenberg and Colquitt (2005) and Colquitt et al. (2001) indicate that such signals particularly affect procedural and interactional justice. Folger and Cropanzano (1998) further argue that consistent ethical behavior is interpreted by employees as respect for personal dignity, forming positive justice perceptions.

**H1:** Digital ethics has a positive effect on organizational justice perception.

When organizations effectively implement data protection measures, employees tend to perceive governance processes as fairer because they feel protected and in control of their data. Suh and Han (2003) found that investment in technical security and clear data control mechanisms positively affects justice perceptions. Culnan and Armstrong (1999) argued that giving employees control over their information increases their sense of respect and fair treatment. Similarly, Luo (2002) and Cavusoglu et al. (2015) confirmed that transparency in data security policies reduces information asymmetry and significantly improves procedural justice perceptions.

**H2:** Data protection practices have a positive effect on organizational justice perception.

### 2.3 Organizational justice as a mediating mechanism toward trust

Trust is not formed directly from governance policies but through employees‘ evaluations of procedural and outcome fairness in policy implementation. Mayer et al. (1995) argued that perceptions of organizational integrity, particularly through procedural justice, form the core foundation of trust. This aligns with system justification theory, which suggests that individuals are motivated to perceive existing organizational arrangements as fair and legitimate (Jost et al., 2004). Dirks and Ferrin (2002) confirmed organizational justice as one of the most consistent predictors of trust. Colquitt and Rodell (2011) explained that justice perceptions affect trust through employees' inferences about organizational motives and intentions, consistent with meta-analytic evidence linking justice perceptions to trust and trustworthiness (Colquitt et al., 2007). In digitalized environments, Greenberg and Colquitt (2005) emphasized that procedural justice becomes increasingly important as decisions are automated and direct organizational-employee interaction diminishes.

**H3:** Organizational justice perception has a positive effect on employee trust in the digital transformation system.

### 2.4 Direct antecedents of employee trust

Leadership support in digital transformation is typically expressed through three key behaviors: communicating clear objectives and transformation roadmaps, modeling digital technology use, and consulting employees before implementing changes. These behaviors reduce ambiguity, increase psychological safety, and reinforce trust. Bencsik et al. (2022) confirmed leadership support as a strong predictor of trust in digital transformation contexts. Mayer et al. (1995) argued that trust is formed through employees' assessments of leadership competence, benevolence, and integrity.

**H4:** Leadership support has a positive effect on employee trust in the digital transformation system.

Digital capabilities influence trust through self-efficacy mechanisms. When employees are proficient with digital tools, they have greater control over system interactions, reducing concerns about operational risks, thereby enhancing trust. Bandura (1991) argued that perceived competence directly affects control perceptions and behavior. Compeau and Higgins (1995) confirmed computer self-efficacy promotes positive attitudes toward new information systems. Van Deursen et al. (2016) confirmed digital skills as important factors enabling individuals to interact effectively and safely in digital environments.

**H5:** Employee digital capabilities have a positive effect on trust in the digital transformation system.

When systems operate stably and employees receive adequate support, learning costs and perceived risks decrease, thereby reinforcing trust. Bencsik et al. (2022) confirmed digital readiness as an important antecedent of trust in digital transformation contexts. Venkatesh et al. (2003) indicated that facilitating conditions are important factors promoting system acceptance. Gefen et al. (2003) showed that training and technical support help employees form perceptions of organizational competence and reliability.

**H6:** Organizational digital readiness has a positive effect on employee trust in the digital transformation system.

### 2.5 DT intensity and organizational culture support

When organizations commit strongly to digital transformation at the strategic level, norms of innovation, continuous learning, and readiness for change gradually form and consolidate. Bindel Sibassaha et al. (2025) confirmed digital transformation as a strong predictor of innovation-supportive culture in banking. Schein (2010) argued that new technology drives organizations to redefine working methods and core values. From the Dynamic Capabilities perspective, Teece (2007) argued that organizations need to continuously reconfigure resources to adapt to changing business environments. Vial (2019) argued that digital transformation not only creates technological changes but also transforms internal organizational values and coordination modes.

**H7:** Organizational DT intensity has a positive effect on organizational culture support.

### 2.6 Trust and organizational culture as dual behavioral channels

Trust reduces risk perception—the primary driver of resistance according to Rogers (1995)—and activates reciprocal behavior per Blau (1964): employees who trust the organization respond with active participation and compliance with digital policies. Oreg (2003) showed that among predictors of resistance to change, distrust in the organization and leadership is the strongest and most consistent predictor across contexts, a pattern further supported by evidence that leadership behavior directly shapes employees' emotional and cognitive reactions to organizational change (Oreg and Berson, 2011). Venkatesh and Davis (2000) confirmed trust as a psychological antecedent of system usage intention, particularly for systems handling sensitive personal data.

**H8a:** Employee trust has a positive effect on adaptive behavior toward digital transformation.

**H8b:** Employee trust has a negative effect on resistance behavior toward digital transformation.

Organizational culture support influences adaptive behavior through group norm mechanisms. When innovation and adaptation are viewed as shared organizational values, employees tend to adjust their behavior to conform to collective expectations. Bandura (1991) argued that cultural norms guide behavior through self-regulation processes, while Schein (2010) and Scott and Bruce (1994) emphasized that innovation-supportive cultural environments are important prerequisites for promoting employee adaptive and innovative behavior.

**H9a:** Organizational culture support has a positive effect on adaptive behavior toward digital transformation.

**H9b:** Organizational culture support has a negative effect on resistance behavior toward digital transformation.

### 2.7 The moderating role of digital capabilities

Beyond its direct effect on trust, digital capability may moderate the relationship between leadership support and trust, because employees with high digital capabilities typically receive and translate technical support from supervisors into system trust more effectively (Bandura, 1991; Compeau and Higgins, 1995; Agarwal and Prasad, 1999). However, this role is not always consistent. In high power-distance contexts, leadership may influence primarily through relational and symbolic signals rather than technical guidance, potentially diminishing the moderating significance of digital capability, consistent with findings that individual differences such as gender and experience shape technology-acceptance pathways in distinct ways (Venkatesh and Morris, 2000; Tan and Lim, 2009). Moreover, digital capability is not equivalent to the ability to process social and organizational signals — the mechanism central to trust formation (Van Deursen et al., 2016), reflecting the broader insight that technology-driven organizational change unfolds through an ongoing, improvisational process rather than a fixed implementation sequence (Orlikowski and Hofman, 1997).

**H10:** Employee digital capabilities moderate the relationship between leadership support and trust in the digital transformation system.

### 2.8 Conceptual framework

**Figure 1:**
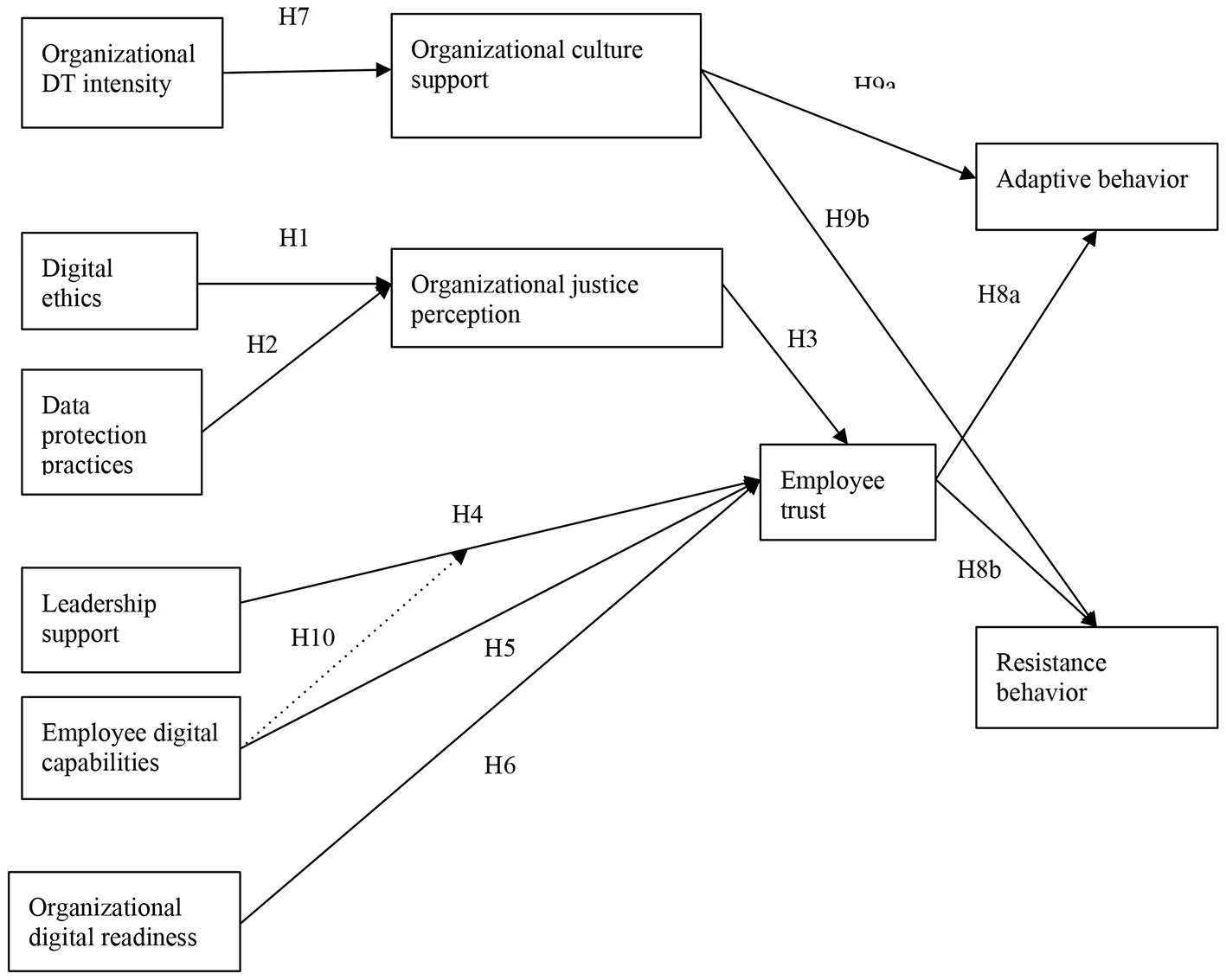
Conceptual framework of the integrated model

Figure 1 summarizes the full set of hypothesized relationships discussed above, showing the two governance antecedents feeding organizational justice, the direct and mediated paths to trust, the parallel DT intensity to organizational culture pathway, the dual behavioral outcomes, and the non-significant moderation path (H10).

## 3 Research methodology

### 3.1 Research design and sampling strategy

The study employs a quantitative cross-sectional design appropriate for testing structural models with multiple latent constructs. PLS-SEM was implemented via SmartPLS (Version 4.0, SmartPLS GmbH, Bönningstedt, Germany; https://www.smartpls.com) (Ringle et al., 2022) and selected over CB-SEM because the integrated model draws on multiple theoretical streams, the sample does not ensure multivariate normality assumptions, and the research objectives include both prediction and structural testing (Hair et al., 2022).

The target population consists of full-time employees at Vietnamese commercial banks who regularly interact with digital systems in their work. Three bank ownership types were purposively included: state-owned commercial banks with high institutional compliance pressures; private joint-stock banks with greater competitive pressure and faster digitization pace; and foreign banks with more mature data governance systems. This diversity enables identification of the consistency of trust formation mechanisms across different institutional environments. A purposive stratified sampling method ensured representation by ownership type, functional department, and geography (Hanoi, Ho Chi Minh City, and Da Nang). Data collection was conducted from January to April 2026. Of 450 questionnaires distributed, 402 valid responses were analyzed after removing 18 inadequate responses, achieving a valid response rate of 89.3% and exceeding the minimum threshold of 10 times the maximum number of paths leading to any latent variable (Hair et al., 2022).

Respondent access was obtained through the human-resources departments of nine partner banks (four state-owned, three joint-stock, two foreign), which distributed the survey link internally to employees who use digital work systems on a regular basis; no external panel or convenience-intercept method was used. Quotas for ownership type, department, and city were set to approximate, though not exactly replicate, the sector's actual workforce composition reported by the Vietnam Banks Association (2023): state-owned banks account for roughly 40–45% of banking-sector employment nationally, joint-stock banks for 40–45%, and foreign banks for the remaining 10–15%, a distribution closely mirrored by the achieved sample (42.0%, 44.8%, and 13.2% respectively). Departmental quotas were set less strictly, since no single published benchmark for functional-department distribution across the sector exists; the resulting overrepresentation of IT staff (22.1% of the sample) relative to typical front-office-heavy bank staffing is acknowledged as a limitation in Section 7.

### 3.2 Sample characteristics

Table 1 summarizes the sample demographic characteristics. Male respondents comprised 54.7% of the sample and females 45.3%. The 31–35 age group was the largest (29.4%), followed by 26–30 years (27.6%). Regarding education, bachelor's degree holders comprised 54.0% and master's degree holders 31.3%. Customer service employees comprised 28.1%, followed by operations (24.1%), IT (22.1%), finance and accounting (15.9%), and HR/training (9.7%). Private joint-stock banks comprised 44.8% of the sample, state-owned banks 42.0%, and foreign banks 13.2%.

**Table 1:** Sample demographic characteristics (*N* =402).

Characteristic	Category	Frequency (%)
Gender	Male	220 (54.7%)
	Female	182 (45.3%)
Bank type	State-owned commercial bank	169 (42.0%)
	Joint-stock commercial bank	180 (44.8%)
	Foreign bank	53 (13.2%)
Education	College/vocational	35 (8.7%)
	Bachelor's degree	217 (54.0%)
	Master's degree	126 (31.3%)
	Doctoral/Other	24 (6.0%)
Tenure	Under 2 years	47 (11.7%)
	2–4 years	101 (25.1%)
	5–8 years	131 (32.6%)
	9–15 years	89 (22.1%)
	Over 15 years	34 (8.5%)

Authors' survey results, 2026.

### 3.3 Measurement instrument and common method bias control

All latent variables were measured using reflective scales with five-point Likert responses (1 = Strongly Disagree; 5 = Strongly Agree). Digital ethics (DE, four items) and data protection practices (DPP, four items) were adapted from Shaikh et al. (2025) with adjustments for Decree 13/2023/ND-CP. Abbreviations are used consistently throughout: POF, retained from the original scale source, denotes organizational justice perception and the two terms are used interchangeably. Leadership support (LS, four items) and digital readiness (DR, four items) were drawn from Bencsik et al. (2022). DT intensity (DT, four items) and organizational culture support (OCS, four items) from Bindel Sibassaha et al. (2025). Adaptive behavior (Adapt, four items) from Scott and Bruce (1994), resistance behavior (Resist, four items) from Oreg (2003), digital literacy (DL, three items) from Van Deursen et al. (2016), organizational justice perception (POF, four items) from Greenberg and Colquitt (2005), and trust (Trust, four items) from Gefen et al. (2003). The questionnaire was translated from English to Vietnamese using independent back-translation, then piloted with 30 bank employees.

Common method bias was controlled at both procedural and statistical levels. Procedurally, respondent anonymity was ensured, item order was randomly reversed, and psychological separation between construct groups was implemented. Statistically, Harman's single-factor test revealed that the largest single factor explained 32.4% of variance, below the 50% threshold, confirming that common method bias is not a critical concern.

### 3.4 Data analysis

PLS-SEM analysis followed the two-step procedure of Hair et al. (2022). Step one evaluated the measurement model through: internal consistency reliability (Cronbach's alpha and composite reliability CR, threshold > 0.70); convergent validity (loadings > 0.70; AVE > 0.50); and discriminant validity via Fornell-Larcker criteria (Fornell and Larcker, 1981) and HTMT indices (Henseler et al., 2015). Step two evaluated the structural model through path coefficients, *t*-statistics, and *p*-values from bootstrapping with 5,000 subsamples.

## 4 Results

### 4.1 Measurement model evaluation

Table 2 presents the measurement model results. All 11 constructs achieved internal consistency reliability with Cronbach's alpha ranging from 0.782 to 0.859 and CR from 0.873 to 0.904, all exceeding the 0.70 threshold. AVE values for all factors ranged from 0.655 to 0.702, all exceeding 0.50, confirming convergent validity. All factor loadings ranged from 0.770 to 0.852, exceeding the 0.70 threshold.

**Table 2:** Measurement model evaluation results.

Construct	Items	Cronbach α	CR	AVE	Loadings
Digital ethics (DE)	4	0.859	0.904	0.702	0.830–0.849
Data protection practices (DPP)	4	0.824	0.884	0.655	0.780–0.822
Leadership support (LS)	4	0.843	0.895	0.680	0.789–0.844
Digital readiness (DR)	4	0.835	0.890	0.669	0.803–0.836
DT intensity (DT)	4	0.826	0.884	0.657	0.770–0.848
Organizational justice (POF)	4	0.841	0.894	0.677	0.800–0.851
Employee trust (Trust)	4	0.841	0.894	0.678	0.814–0.841
Org. culture support (OCS)	4	0.835	0.890	0.668	0.786–0.837
Adaptive behavior (Adapt)	4	0.840	0.892	0.675	0.783–0.843
Resistance behavior (Resist)	4	0.827	0.885	0.658	0.794–0.852
Digital literacy (DL)	3	0.782	0.873	0.696	0.831–0.839

CR, composite reliability; AVE, average variance extracted. Thresholds: alpha > 0.70; CR > 0.70; AVE > 0.50; loadings > 0.70 (Hair et al., 2022).

Discriminant validity was assessed via the heterotrait-monotrait ratio of correlations (HTMT; Henseler et al., 2015) (Table 3). All HTMT values remained below the conservative threshold of 0.85, confirming discriminant validity across the model. The highest observed ratio was 0.661, between DT intensity and organizational culture support, consistent with their theoretical proximity as sequential organizational-level constructs; the second highest was 0.625, between organizational justice and trust; all remaining ratios fell below 0.60.

**Table 3:** Heterotrait–monotrait Ratio (HTMT) for discriminant validity assessment.

Adapt	DE	DL	DPP	DR	DT	LS	OCS	POF	Trust
0.538									
0.446	0.471								
0.407	0.582	0.297							
0.416	0.432	0.573	0.400						
0.526	0.406	0.513	0.338	0.453					
0.536	0.435	0.451	0.492	0.586	0.452				
0.540	0.421	0.510	0.299	0.416	0.661	0.469			
0.543	0.538	0.398	0.467	0.364	0.373	0.397	0.492		
0.590	0.620	0.516	0.425	0.546	0.446	0.582	0.536	0.625	

### 4.2 Structural model and hypothesis testing

Table 4 summarizes the results of testing all ten hypotheses (twelve directional paths, following consolidation of the symmetric adaptive/resistance-behavior effects into H8 and H9), with eleven paths supported and H10 rejected.

**Table 4:** Structural model results and hypothesis testing.

H	Path	Beta	SE	*t*	*p*	*f* ^2^	Decision
H1	DE → POF	0.355	0.048	7.379	0.000	0.127	Supported^***^
H2	DPP → POF	0.215	0.050	4.341	0.000	0.047	Supported^***^
H3	POF → Trust	0.349	0.040	8.675	0.000	0.181	Supported^***^
H4	LS → Trust	0.241	0.042	5.787	0.000	0.073	Supported^***^
H5	DL → Trust	0.138	0.050	2.780	0.006	0.025	Supported^**^
H6	DR → Trust	0.171	0.046	3.714	0.000	0.035	Supported^***^
H7	DT → OCS	0.554	0.033	16.828	0.000	0.443	Supported^***^
H8a	Trust → Adapt	0.372	0.046	8.146	0.000	0.161	Supported^***^
H8b	Trust → Resist	−0.371	0.043	8.680	0.000	0.153	Supported^***^
H9a	OCS → Adapt	0.287	0.046	6.303	0.000	0.096	Supported^***^
H9b	OCS → Resist	−0.245	0.047	5.209	0.000	0.067	Supported^***^
H10	LS × DL → Trust	0.003	0.033	0.107	0.915	0.000	Not Supported

^***^p < 0.001; ^**^p < 0.01; ns = not statistically significant. Bootstrapping with 5,000 subsamples. *R*^2^ (Trust) = 0.439; *R*^2^ (POF) = 0.247; *R*^2^ (OCS) = 0.307. *R*^2^ (Adapt) = 0.316 (adjusted 0.313); *R*^2^ (Resist) = 0.279 (adjusted 0.276); adjusted *R*^2^ (Trust) = 0.432; adjusted *R*^2^ (POF) = 0.243; adjusted *R*^2^ (OCS) = 0.305. All inner-model VIF values ranged from 1.00 to 1.52, well below the conservative threshold of 3.0 (Hair et al., 2022), indicating collinearity is not a concern. *Q*^2^ values from blindfolding (omission distance = 7) were 0.163 (POF), 0.291 (Trust), 0.201 (OCS), 0.208 (Adapt), and 0.180 (Resist), all above zero and confirming predictive relevance for every endogenous construct.

For justice perception antecedents (*R*^2^ = 0.247), digital ethics is the stronger predictor (β = 0.355; *t* = 7.379; *p* < 0.001) compared to data protection practices (β = 0.215; *t* = 4.341; *p* < 0.001), supporting H1 and H2. Both factors are highly significant, indicating that responsible digital governance requires both ethical intent and technical data protection capability.

For trust antecedents (*R*^2^ = 0.439), organizational justice is the strongest predictor (β = 0.349; *t* = 8.675; *p* < 0.001), confirming H3 and its important mediating role. Leadership support (β = 0.241; *t* = 5.787), digital readiness (β = 0.171; *t* = 3.714), and digital literacy (β = 0.138; *t* = 2.780) all have significant direct effects, confirming H4, H5, and H6.

For organizational culture support (*R*^2^ = 0.307), DT intensity has the largest path coefficient in the entire model (β = 0.554; *t* = 16.828; *p* < 0.001), confirming H7. For behavioral outcomes, trust promotes adaptation (β = 0.372) and reduces resistance (β = −0.371) with nearly symmetric coefficients, confirming H8a and H8b. Organizational culture promotes adaptation (β = 0.287) and reduces resistance (β = −0.245), confirming H9a and H9b.

#### 4.2.1 Moderation path (H10) and its non-significance

H10, testing the moderating effect of digital capabilities on the leadership support–trust relationship, yielded results near zero and statistically non-significant (β = 0.003; *t* = 0.107; *p* = 0.915). This is a clear rejection with important theoretical implications discussed in the following section.

### 4.3 Mediation analysis: indirect effects on trust

Following the reviewer's request for formal mediation testing, bootstrapped indirect effects (5,000 subsamples) were estimated for the two paths through which digital ethics governance and data protection practices are theorized to influence trust via organizational justice perception. Table 5 reports the results.

**Table 5:** Bootstrapped indirect effects (mediation via organizational justice).

Indirect Path	Indirect β	SE	*t*	*p*	95% CI
DE → POF → Trust	0.124	0.024	5.13	0.000	[0.078, 0.171]
DPP → POF → Trust	0.075	0.020	3.82	0.000	[0.038, 0.115]

Bias-corrected bootstrap confidence intervals (5,000 resamples); neither interval contains zero, confirming mediation via organizational justice in both paths, consistent with the significant direct effects of DE and DPP already reported in Table 4.

Both indirect effects are statistically significant and their confidence intervals exclude zero, confirming that organizational justice perception mediates the effect of digital ethics and data protection practices on employee trust. Because the direct paths DE → POF and DPP → POF (Table 4) and POF → Trust (H3) are all significant, and the indirect effects reported here are also significant, both relationships are classified as complementary (partial) mediation rather than full mediation, indicating that digital ethics and data protection practices retain the capacity to shape trust through channels beyond perceived organizational justice.

### 4.4 Robustness check: PLSpredict analysis

To address the reviewer's request for a robustness test, an out-of-sample predictive analysis was conducted using the PLSpredict procedure (Shmueli et al., 2019) with 10-fold cross-validation (*k* = 10, 10 repetitions). Root-mean-square error (RMSE) values for each of the 20 reflective indicators of the five endogenous constructs (Organizational Justice, Trust, Organizational Culture Support, Adaptive Behavior, and Resistance Behavior) were compared against a naive linear-regression (LM) benchmark.

Of the 20 indicators, 18 showed lower PLS-SEM RMSE than the LM benchmark; the only two exceptions were the organizational culture support indicators OCS1 and OCS4, for which PLS and LM RMSE were nearly identical (differences under 0.03). Following the decision rules of Shmueli et al. (2019), this large majority of indicators with lower PLS-SEM prediction error relative to the naive benchmark indicates medium-to-high predictive power for the model as a whole. Combined with the *Q*^2^ values reported in Table 4, this provides converging evidence that the structural model has genuine out-of-sample predictive relevance rather than merely in-sample explanatory fit.

## 5 Discussion

### 5.1 Governance-to-justice path (H1, H2)

The results show that digital ethics (β = 0.355) exerts a stronger effect on justice perceptions than data protection practices (β = 0.215), though both are highly significant. This difference reflects a fundamental distinction in how employees experience data governance. While data protection practices represent pure organizational capability—present in technical infrastructure, control systems, and operational procedures—digital ethics represents organizational will, manifested through public commitments, symbolic leadership behavior, and moments when organizations proactively explain why employee data is used in particular ways. Employees thus perceive the organization not as a neutral technical system but as an intentional moral agent. In Vietnam's high power-distance context, employees are particularly sensitive to signals about organizational values and intentions through symbolic leadership actions, consistent with organizational symbolism theory (Pfeffer, 1981). This finding extends the work of Shaikh et al. (2025), where both factors had more balanced effects, suggesting the relative contribution of ethical versus technical factors to justice perception may be culturally contingent rather than a theoretical constant.

### 5.2 Justice-to-trust path (H3) and direct trust antecedents (H4–H6)

Organizational justice emerged as the strongest predictor of trust among direct antecedents (β = 0.349; *R*^2^ = 0.439), reinforcing the theoretical proposition that trust is not formed directly from digital governance policies but is constructed through employees' evaluations of the fairness of policy implementation. Employees trust the organization not because it declares commitment to digital ethics or has data protection policies, but because they perceive these policies to be implemented consistently, transparently, with respect for personal dignity, and without discrimination. As digitalization deepens and automated decisions reduce direct organizational-employee interactions, organizational justice becomes a particularly crucial mechanism for employees to evaluate organizational transparency, accountability, and goodwill. This finding aligns with Greenberg and Colquitt (2005), Mayer et al. (1995), and Dirks and Ferrin (2002) on justice as the foundation of trust, while adding the perspective of organizational digitalization as a condition that amplifies the central role of justice perceptions.

The clear rejection of H12 (β = 0.003; *t* = 0.107; *p* = 0.915) constitutes a theoretically notable finding. Digital capability does not significantly modify the effect of leadership support on trust, implying that leadership influence does not operate primarily through technical knowledge transfer or capability building. Instead, in Vietnam's high power-distance management culture, employees tend to interpret leadership signals as assurances about the legitimacy, fairness, and safety of the change process rather than as purely technical expertise evaluations. When leaders demonstrate clear commitment to data privacy, maintain consistency between statements and actions, and are willing to listen to employee concerns about new digital systems, trust forms relatively stably regardless of employee digital capability levels. This suggests that in the Vietnamese banking context, leaders function not merely as change managers but as ethical guarantors for the entire digital transformation process.

### 5.3 Organizational-context path (H7, H8a/b, H9a/b)

Finally, the study presents a noteworthy theoretical extension of the Privacy Calculus Theory. According to this theory, individuals are assumed to be autonomous agents deciding on information disclosure based on benefit-risk calculations (Dinev and Hart, 2006). However, evidence from this study suggests that in the Vietnamese banking context, this calculation is not entirely individual. The strongest predictor of organizational culture support is organizational-level DT intensity (β = 0.554), while organizational culture subsequently influences both employee adaptation and resistance. Employees do not merely weigh privacy costs at the individual level but respond to collective norms, group expectations, and institutional pressures surrounding them. Individually-oriented privacy models—such as informed consent frameworks—may therefore need adjustment when applied in collectivist cultural contexts where privacy is not solely an individual choice but carries clear social and relational dimensions.

## 6 Managerial implications

Four managerial directions are proposed for Vietnamese commercial banks and digital policy-makers.

First, establish a transparent and accountable employee data governance framework. Banks should issue internal regulations specifying which data may be collected, usage purposes, retention periods, authorized access, and violation handling mechanisms. Organizations must commit not to use personal data beyond work purposes or for monitoring activities exceeding previously communicated scopes. Data governance transparency not only ensures compliance with Decree 13/2023/ND-CP but contributes to consolidating employee trust in digital transformation programs.

Second, establish employee consultation and consent mechanisms for decisions involving internal data. Rather than top-down data governance approaches, banks should create conditions for employees to access information, receive explanations, and provide feedback on data collection, storage, or usage policies. Decisions involving digital monitoring systems, algorithmic performance evaluation, or HR data analysis should be clearly communicated regarding objectives, scope, and usage limits.

Third, strengthen the role of employee representative mechanisms in data governance oversight. Unions or employee representative organizations should be empowered to review, critique, and monitor the implementation of internal data policies, creating an independent oversight channel that enhances transparency, reduces information asymmetry, and provides protection when privacy-related disputes arise.

Fourth, shift the focus of digital transformation from technology governance to trust governance. Employee trust is formed not only from technical capability or digital infrastructure but is significantly influenced by how organizations implement data policies and treat employees. Banks must build governance principles based on fairness, accountability, and respect for employee privacy. Only when employees trust that their data is used legally, transparently, and responsibly will digital transformation programs receive sustainable internal cooperation.

## 7 Conclusion

This study provides empirical evidence on factors influencing employee trust toward digital transformation systems in Vietnamese commercial banks through an integrated model connecting digital ethics, organizational justice perceptions, and adaptive behaviors. The findings confirm most proposed hypotheses, showing that employee trust is formed not only from digital ethics governance and data protection practices but is also influenced by leadership support, individual digital capabilities, and organizational digital readiness. The study affirms the central role of organizational justice perception as an important psychological mediating mechanism between governance practices and employee trust. Additionally, DT intensity contributes to promoting organizational culture support, while trust and organizational culture serve as dual primary behavioral channels influencing employee adaptation and resistance to digital transformation.

The study contributes theoretically by integrating the Resource-Based View, Organizational Climate Theory, Social Exchange Theory, Privacy Calculus Theory, and Technology Acceptance Model in a unified analytical framework, while providing empirical evidence from the commercial banking context of a developing economy. Managerially, the results emphasize that digital transformation success depends not only on technology investment but requires a fair, transparent organizational environment with accountability in data governance and consistent leadership support.

Limitations include cross-sectional design, self-reported data, and sample scope. The IT staff overrepresentation (22.1%) may affect trust perceptions and resistance behavior findings. Future research should examine nonlinear or threshold effects of DT intensity, conduct multi-group analyses across technical and business units, longitudinal surveys, and cross-country comparisons to clarify the role of organizational and cultural context in trust formation under digital transformation.

## Data Availability

The raw data supporting the conclusions of this article will be made available by the authors, without undue reservation.
